# Flexibility within the Rotor and Stators of the Vacuolar H^+^-ATPase 

**DOI:** 10.1371/journal.pone.0082207

**Published:** 2013-12-02

**Authors:** Chun Feng Song, Kostas Papachristos, Shaun Rawson, Markus Huss, Helmut Wieczorek, Emanuele Paci, John Trinick, Michael A. Harrison, Stephen P. Muench

**Affiliations:** 1 Electron Microscopy Center, Hebei Medical University, Shijiazhuang, China; 2 School of Molecular and Cellular Biology, University of Leeds, Leeds, United Kingdom; 3 School of Biomedical Sciences, University of Leeds, Leeds, United Kingdom; 4 Abteilung Tierphysiologie, Fachbereich Biologie/Chemie, Universität Osnabrück, Osnabrück, Germany; University of Edinburgh, United Kingdom

## Abstract

The V-ATPase is a membrane-bound protein complex which pumps protons across the membrane to generate a large proton motive force through the coupling of an ATP-driven 3-stroke rotary motor (V_1_) to a multistroke proton pump (V_o_). This is done with near 100% efficiency, which is achieved in part by flexibility within the central rotor axle and stator connections, allowing the system to flex to minimise the free energy loss of conformational changes during catalysis. We have used electron microscopy to reveal distinctive bending along the V-ATPase complex, leading to angular displacement of the V_1_ domain relative to the V_o_ domain to a maximum of ~30°. This has been complemented by elastic network normal mode analysis that shows both flexing and twisting with the compliance being located in the rotor axle, stator filaments, or both. This study provides direct evidence of flexibility within the V-ATPase and by implication in related rotary ATPases, a feature predicted to be important for regulation and their high energetic efficiencies.

## Introduction

The vacuolar H^+^-ATPases (V-ATPase) and the related F_1_F_o_-ATPases (F-ATPase) are large membrane-bound complexes that are highly efficient energy conversion machines [1,2]. In the V-ATPases, the free energy of ATP hydrolysis is used to move protons across the membrane against an electrochemical potential [3]. For the F-ATPase operating in synthase mode, the energy associated with the proton motive force is converted to produce ATP. Both proton pumping and ATP synthesis use a rotational mechanism [4,5].

**Figure 1:**
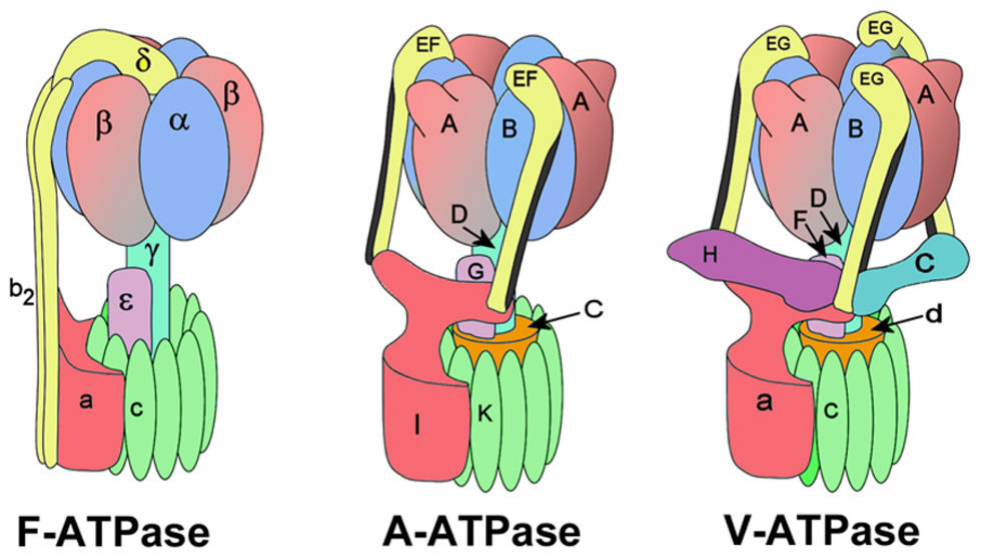
Subunit organisation in the rotary ATPase family. In the V-ATPase, the D/F/d/c-ring structure is the rotor, (AB)_3_/(EG)_3_/C/H/*a* is the stator. Equivalent subunits within each complex are in the same colour.

 In V-ATPase, ATP hydrolysis occurs in the soluble V_1_ domain where 3 subunit A/B catalytic dimers function cooperatively, with their active sites sequentially cycling between open (no nucleotide bound), loose (ADP +Pi bound) and tight (ATP hydrolysing) states, similar to the mechanism suggested for the F-ATPase [6]. This three-step motor applies torque to a central rotor axle comprising subunits D and F, which in turn connects to a ring of proton-translocating integral membrane c subunits via a ‘socket’ built by the d subunit [4]. Torque on the axle drives rotation of the c-ring [7,8]. By analogy to the F_1_F_o_ ATPase, proton translocation is proposed to occur at the transient interface formed between the external surface of the rotating c-ring and the single copy membrane subunit *a* [9,10]. To avoid subunit *a* co-rotating with the c-ring, it is linked to V_1_ by a stator network (Figure 1). The structures of the yeast and tobacco hornworm (*Manduca sexta*) V-ATPases from single particle cryo-EM have revealed that the stator network consists of 3 subunit E/G heterodimers connected to subunits C, H and the cytoplasmic N-terminal domain of subunit *a* (a_N_) between the V_1_ and V_o_ domains [11–13] (Figure 1). A much simpler arrangement is present in the F-ATPase, where only 1 stator filament joins the proton translocating F_o_ and ATP synthesising F_1_ domains (Figure 1) [14]. The bacterial A-ATPase, which in different organisms can operate as either a H^+^ or Na^+^ pump and ATP synthase, has been shown to be more complex than the F-ATPase, but simpler than the eukaryotic V-ATPase, with 2 stator filaments [12,15,16]. 

 A common feature of rotary ATPases is a symmetry mismatch where the 3-stroke motor in the F_1_/A_1_/V_1_ domain is linked to a c-ring in F_o_/A_o_/V_o_ that can vary in size. In the A-ATPase it is a decamer [15,17], but in F-ATPase it can have between 8 and 15 subunits and commonly does not contain a multiple of 3 c-subunits [18–20]. In F-ATPase, this variability has been hypothesised to be an adaptation to different prevailing membrane potentials [21,22]. Thus there is no simple relationship between the stepping of the two motors and there must be a way to accommodate the symmetry mismatch. As such, the two functions of the ATPase are linked only by the torque of the rotor, applied continually by ATP hydrolysis steps (when ATP is not limiting) in the case of the V- and A-ATPase, or by individual proton translocation steps in the ATP synthase. A possible reason for this symmetry mismatch would be to avoid the deeper energy minima that would result from a matched symmetry, whereby one ATP is not directly linked to one proton translocation but indirectly to multiple transport processes, not always of an integer stoichiometry value [23]. By avoiding deep energy minima, these systems can function at much higher efficiencies. An extension to this is the presence of energy-storing elastic linkages capable of buffering energy transfer between soluble and membrane domains, negating the need for synchronously co-ordinated stepping of the ATPase and proton pump motors. Such elasticity could be in the central rotor axle or the stator. In rotary ATPases, these must also have sufficient flexibility to accommodate the large conformational changes that occur in the soluble head domains during generation of rotation [24]. 

 Crystal structures have suggested flexibility in the F-ATPase where the rotor axle connects to F_o_ [25–27]. In general though, crystal structures give only a snapshot of a static, low energy state and are unable to show to what level the stator can resist flexing. However, crystal lattice constraints are not a factor in EM studies of single particles. These have also suggested flexibility in the rotor axle [28–30]. Normal mode analysis of the stators in the A-ATPase has shown that these can accommodate flexing in the radial direction that could accommodate ‘wobble’ occurring during the catalytic cycle [31]. Understanding flexibility in these systems is likely to be an important step towards mechanical power transmission at near-100% efficiency in these rotary motors. 

 The V-ATPase is regulated in some cells by a dissociation mechanism, whereby V_1_ dissociates from V_o_ during times of energy depletion, for example glucose depletion (in *Saccharomyces cerevisiae*) or insect larval moulting (in *Manduca sexta*) [32,33]. The structural changes which accompany the dissociation process remain poorly understood but the phosphorylation of subunit C is predicted to trigger the process [34–36]. Although the structure of the dissociated *Manduca* V_1_ domain has revealed the conformational changes that occur after separation, the processes that lead to dissociation remain poorly resolved [37]. However, tomography and electron crystallography studies on the *Thermus thermophilus* A-ATPase have shown large angle flexing along the long axis linking soluble and membrane domains after changes in pH and temperature that trigger dissociation [38]. 

 Here we present evidence for flexibility of the yeast and *Manduca* V-ATPases using negative stain and single particle cryo-EM in combination with single particle averaging and classification techniques. This has been combined with normal mode analysis of an elastic coarse-grained model of the V-type ATPase holoenzyme. Together these approaches allow exploration of the idea that V-ATPases (and by implication other rotary ATPases), rather than being rigid, have inherent flexibility that is likely to contribute to their remarkably high efficiency and may play an important role in controlled dissociation.

## Materials and Methods

### Protein extraction and purification

Yeast V-ATPase was obtained using EDTA-washed vacuolar membrane vesicles prepared as previously described [39] from the haploid strain W303-1B. Cells were grown to mid-log phase on yeast extract-peptone medium containing 2% glucose. Isolated membrane vesicles were resuspended to a protein concentration of 4 mg ml^-1^ in 10mM Tris-HCl pH 7.5, 0.1mM EDTA, 10% glycerol. Buffers were supplemented with PMSF (1 mM) and a protease inhibitor cocktail (Roche). To the membrane suspension on ice, 30% (w/v) dodecyl maltoside (DDM) was added drop-wise whilst continually stirring to a yield a final detergent:protein ratio of 10:1 (w/w). The membranes were stirred for a further 30 minutes on ice and centrifuged at 100,000g for 1 hour at 4°C. The supernatant was applied to a Superose 6HR column (Amersham Biosciences) equilibrated with 50mM Tris-HCl pH 7.5 containing 5mM MgCl_2_, 20% glycerol, 1mM DTT and 0.15% (w/v) DDM and eluted with the same buffer at a flow rate of 0.25ml/min. Fractions (1 ml) were assayed for ATPase activity as described in [40], those containing activity were pooled and concentrated using a centrifugal concentrator with 100 kDa cut-off filter (Centricon). *Manduca* V-ATPase was extracted and purified as previously described [41,42].

### Electron microscopy and image processing

Carbon coated grids were placed under a UV lamp for 40 minutes prior to use and ~3µl of protein solution (~50µg/ml) placed onto a carbon-coated grid before staining with 1% uranyl acetate [43]. Images of yeast and *Manduca* V-ATPase were taken on a Jeol 1200EX microscope fitted with a LaB_6_ filament and operating at 80 kV and 40k magnification. Micrographs were recorded on Kodak So 163 film, and scanned using a NIKON Coolscan scanner with a resulting pixel size of 4.4Å/pixel. The *Manduca* Malpighian tubule V-ATPase both in the absence and presence of 5mM Mg.ATP was imaged on a FEI F20 microscope at a magnification of 69,000 with a Gatan 4k x 4k CCD camera. The resulting pixel size was 2.18Å/pixel.

**Figure 2:**
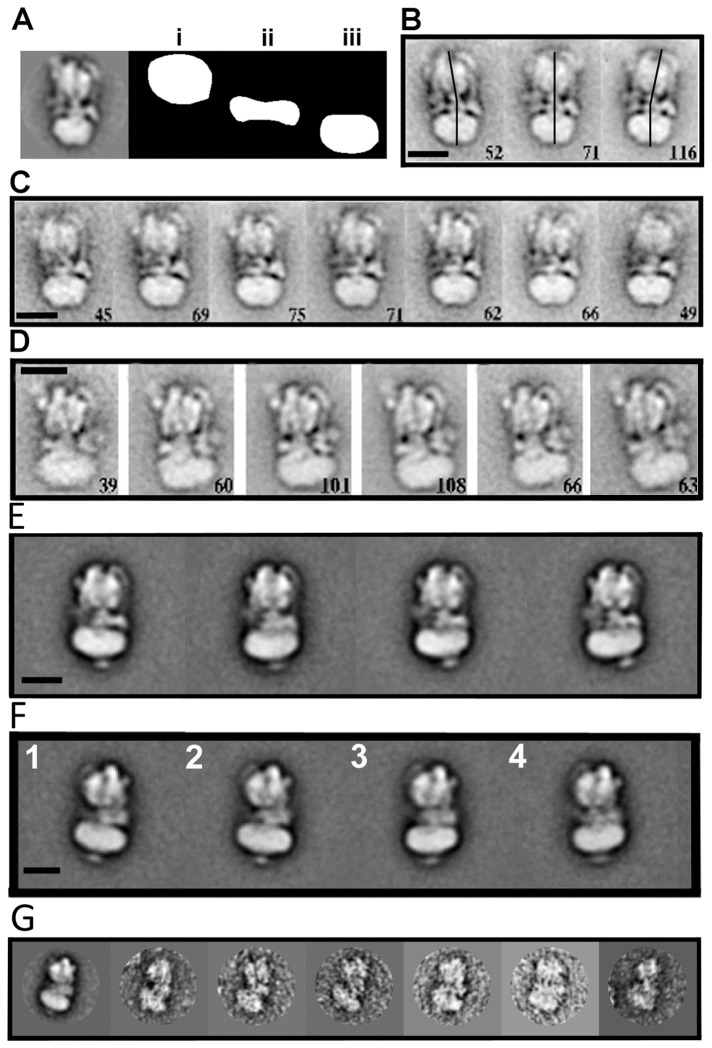
Negative stain electron microscopy of the *Saccharomyces* and *Manduca* *sexta* V-ATPases. (A) A representative class of yeast V-ATPase alongside the 3 masks used to extract the V_1_ (i), central (ii) and V_o_ domains (iii). (B, C) Yeast V-ATPase classes of particles belonging to the same orientation, as determined from multi-reference alignment, re-aligned to V_o_ and classified using a mask over V_1_. (D) Yeast V-ATPase particles of particular views aligned against V_1_ and classified by V_o_. Numbers in the bottom right corner of B-D, are particle numbers in each class. (E, F) *M*. *sexta* V-ATPase classes of particles belonging to the same orientation and aligned against V_1_ and classified around V_o_. (G) *M*. *sexta* V-ATPase class (far left) and representative views of some of the particles making up the class. In all cases scale bars represent 120Å.

Particles were picked using BOXER and then normalised and band pass filtered using SPIDER [44,45]. The data were centred, aligned and classified using IMAGIC-5, producing classes which displayed clear structural details including stator connections [46]. Particles which aligned poorly, produced classes of dissociated V-ATPase complex or were unstable during processing were removed leaving 13816 and 16743 particles from *Manduca sexta* and yeast, respectively. The movies of conformational flexibility were generated using the method developed by Burgess and co workers [47]. In the first instance particles were aligned using projections of the *Manduca* V-ATPase model. Particles were then grouped according to the projection to which they aligned. The particles that corresponded to each projection were then re-aligned using only V_1_ which was isolated by masking. The aligned stack was then classified on the basis of the V_o_ domain thus showing variation specifically within this region (Figure 2A). By grouping the particles to specific projections and then sub-classifying each group of particles, artifacts produced by rotational orientation are reduced. This was then repeated by aligning the particle stacks for each projection to just V_o_ and classifying the data set based on the V_1_ domain. In addition, V-ATPase particles were aligned using the whole molecule, with classification conducted on just the central region using the mask shown in Figure 2A. In order to visualise the flexibility, these classes were placed into a gallery and displayed as a movie which highlighted conformational variation. Note that these movies contain the frames in an arbitrary order and were generated with each “conformation” having equal weight. Data were also processed using the full image stack and a large circular mask and the resulting classes inspected to identify those that displayed signs of flexibility (Figure S1). 

 To investigate effects of ATP on flexibility, 460 and 406 micrographs were collected for samples with or without Mg.ATP, respectively. Data were then processed using BOXER in EMAN2 and resulted in 7510 (+ATP) and 7494 (-ATP) particles after the removal of poor particles [48]. Both data sets were subject to multi-reference alignment using the same references, and classification was performed using the same mask in IMAGIC-5 [46]. This ensured that the data were handled and processed in the same manner for both samples. 

 Data collected in a cryo-EM study of *Manduca* V-ATPase were also analysed to test if flexibility by negative staining was exaggerated by the staining method [12]. Cryo EM does not involve staining and data are assumed to not be subject to surface tension or drying forces. Images were recorded at 69,000 x magnification on an FEI F20 microscope fitted with a Gatan 4k x 4k CCD camera. Since cryo-EM allows for a range of orientations within the ice, the side views were extracted by re-projecting the V-ATPase reconstruction and aligning the image stack to the re-projections. Only those particles which aligned to the side view re-projections were then used (3718 particles) for processing. The data were aligned and processed in the same manner as the negative stain data, with the V_1_ being fixed during alignment and classified using a mask around V_o_ and vice versa.

### Modelling

The 3D cryoEM map of intact *Manduca* V-type ATPase [12] was used to obtain a coarse-grained (CG) representation of the ATPase complex. Using a topology-preserving algorithm [49] as implemented in the SCULPTOR [50] visualization software, a pseudo-particle model was constructed consisting of 250 beads. The CG model was then used as a scaffold for a Bend-Twist-Stretch Elastic Rod Network Model (ERNM) [51]. Application of standard Elastic Network Models (ENMs) using coarse-grained representations of EM 3D maps has previously been reported as a means to investigate bio-macromolecular dynamics [52,53] and assist the EM 3D reconstruction process [54]. However, drastic CG is not expected to give accurate EN models, and eigenvectors calculated with these models should be considered more as a reduced-space sub-basis for conformational description. ERNM, on the other hand incorporates elements from basic linear-elasticity theory, providing a more realistic description of protein flexibility. The Hessian matrix and corresponding eigenvectors were calculated using the Python-based script suite MODEHUNTER (http://modehunter.biomachina.org/). The first two non-trivial eigenvectors were used to visualize the intrinsic modes of the intact ATPase. 3D pseudo-maps were reconstructed from various structures along the first two eigenvectors. Due to the synthetic pseudo-maps being noisy, a Gaussian filter was used to smooth them using CHIMERA [55]. These models along with the V-ATPase reconstruction in a “non-deformed” state were re-projected into 50 different views. The re-projections from the three models were then combined into one file and low pass filtered to 35 Å. 

## Results

### Electron microscopy

Initial electron microscopy by negative stain of both the *Manduca* and *Saccharomyces* V-ATPase revealed that the samples were monodisperse with little background noise, or evidence of full dissociation. The negatively stained V-ATPase shows a preferred orientation, lying with its long axis parallel to the carbon support film due to its roughly cylindrical shape such that side views are mainly seen. After alignment and classification of the data, the resulting class averages were well-defined and detailed (Figure 2). 

### Analysis of flexibility in the negative stain data

In order to investigate the flexibility of the connections between V_o_ and V_1_, the image stacks were aligned against V-ATPase projections and separated in accordance to their corresponding projection view. Each view was then aligned to the masked V_o_ domain and then classified using a mask covering only V_1_ (Figure 2A). The resulting classes showed a variety of views, which demonstrated deviation from the axle-c-ring co-axis, consistent with flexing of the linkages between V_1_ and V_o_ (Figure 2B, C). By processing the data in the opposite way, with the image stack belonging to each projection being aligned to V_1_ and with V_o_ used for classification, distinctive classes with V_o_ displaced from the central axis were also observed (Figure 2D). Similar observations were made with negatively-stained *Manduca* V-ATPase aligned for V_1_ and classified around V_o_ (Figure 2E, F). In order to better demonstrate flexibility, classes were placed into a gallery and displayed as movies (Movies S1-S7). Both the *Manduca* and yeast enzymes show a maximum bending of V_o_ relative to V_1_ of ~30° (Figure 2D, F). The whole data set, not separated by their correlation to a specific angular view, was also aligned and classified using a full mask. The also produced a range of classes which showed clear bending along the long axis of the complex (Figure S1).

To determine the proportion of the particles flexing, all 16742 particles in the *Manduca* dataset were re-classified into 490 classes. Analysis of the corresponding classes showed 10915 ‘straight’ particles (75% of the total), 2135 particles where V_1_ flexes away from subunit *a* (14%) and 1641 particles where V_1_ flexes towards subunit *a* (11%). A selection of some of the particles that make up one of these classes is shown in Figure 2G. The ‘straight’ classes may include particles where the plane of flexing is perpendicular to that of the gird, as opposed to parallel to it. This might be observed as apparent shortening of the complex, but would be difficult to identify given the modest resolution of the classes. The yeast data were also re-classified into 300 classes, with 9817 particles (72%) being straight, 2006 (14%) with V_1_ flexing away from subunit *a* and 2001 (14%) with V_1_ flexing towards subunit *a*. The data were also re-aligned using re-projections from 3 models, the ‘normal’ ground state V-ATPase reconstruction, and the 2 extreme cases of flexing suggested by normal mode analysis (discussed below). For both *Manduca* and yeast data, a significant proportion (~74%) aligned best to the non-flexed V-ATPase reconstruction. 

 These independent tests indicate that a substantial population (~25%) of particles in both the yeast and *Manduca* datasets adopt apparently strained conformations. This bending along the long axis of the complex brings V_1_ and V_o_ closer to each other. This percentage may be an underestimation since particles which bend adjacent to the plane would be difficult to identify. Although a significant proportion of the V-ATPase population exists in an apparent “straight” ground state it is clear that a substantial subset deviate from this and may provide mechanistic insight into this complex system. 

### Analysis of flexibility in the cryo-EM data

**Figure 3:**
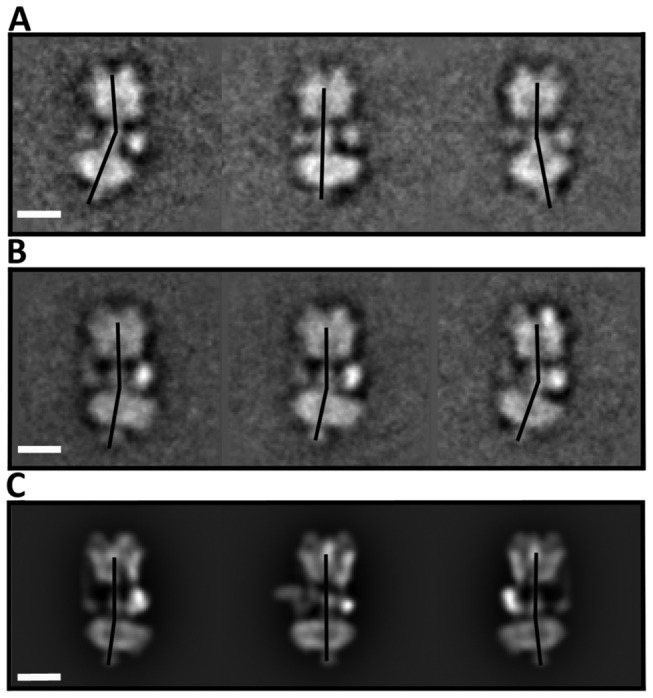
Cryo-EM analysis of Flexibility in *Manduca* V-ATPase. (A, B) Single particle cryo-EM analysis of the *M*. *sexta* V-ATPase enzyme aligned against V_1_ and classified against V_o_. (C) Reprojections from the *M*. *sexta* reconstruction showing the greatest displacement of V_1_ relative to V_o_. Scale bars represent 100Å.

The use of heavy metal stains and carbon substrate can induce artefacts, such as flattening and distortion of particles, although bending towards the plane of the grid and not parallel to it (as observed in Figure 2) appears more likely. In order to investigate this possibility, cryo-EM data for native state *Manduca* V-ATPase in vitreous ice were also analysed. The proportion of side view particles showing flexing (721 of 3718 (~19%)) is slightly lower than that of the negative stain data (25%). The dataset was separated into particular views and then aligned to the V_1_ domain and classified using a mask to the V_o_ domain. Despite the lower contrast of the classes flexing between the 2 domains could be seen with a maximum deviation of ~25° in the long axis of the complex (Figure 3A left panel) and ~20° (Figure 3B right panel) away from subunit *a* (see also Movies S6-S7). Since the V-ATPase cryo-EM particles have much more rotational freedom than those seen in negative stain (where the V-ATPase lies flat against the carbon surface), care must be taken not to confuse flexibility with rotation within the plane of the viewer. In order to judge that the flexing seen was not due to an artefact of viewing from any particular angle, the *Manduca* V-ATPase reconstruction [12] was re-projected using an angular spread of 10°. Analysis of the resulting 418 projections showed no significant flexing between the V_1_ and V_o_ domains (Figure 3C). 

### The influence of ATP on flexibility

**Figure 4:**
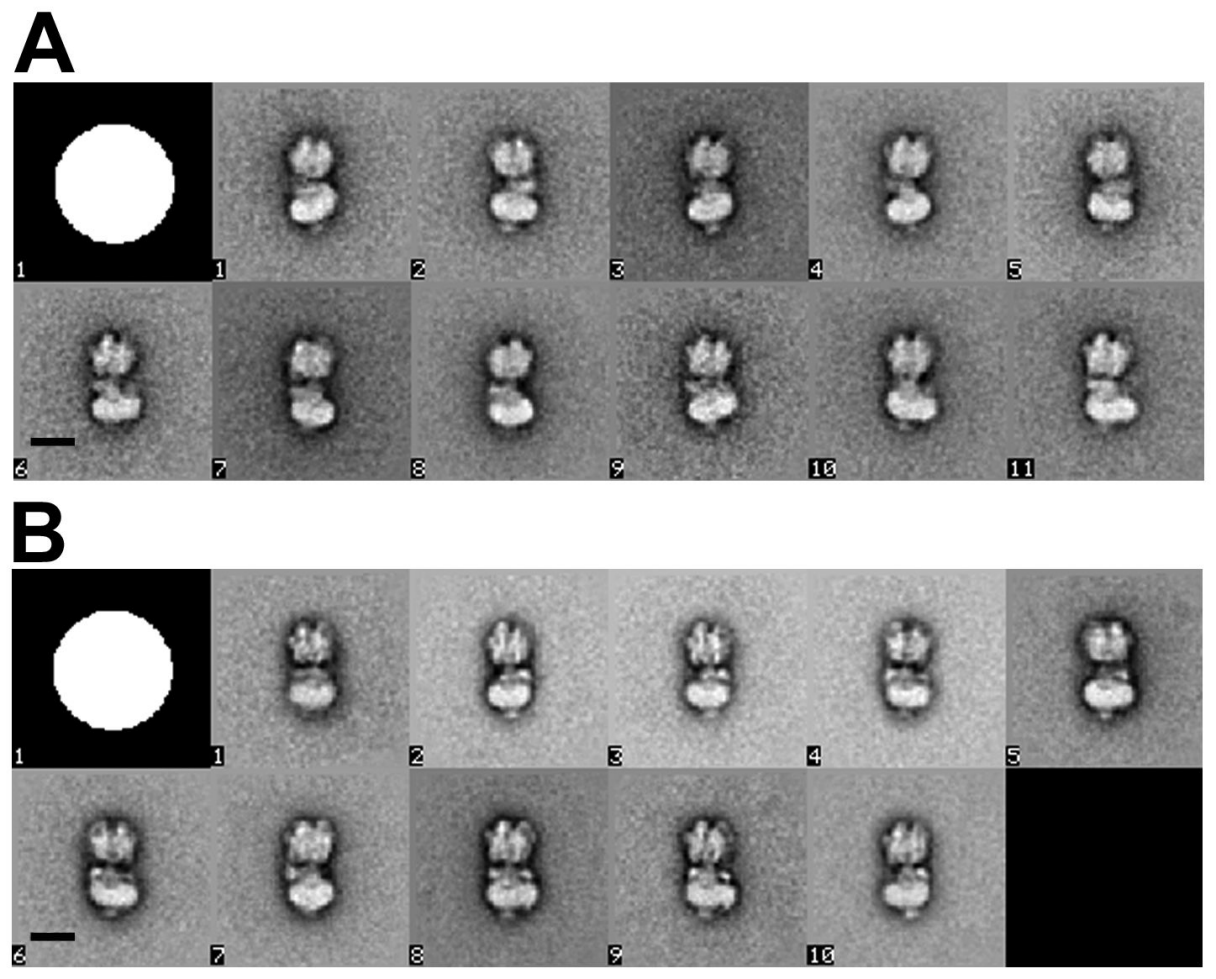
The influence of ATP on V-ATPase flexibility. The classes which represented the most degree of flexing between V_1_ and V_o_ for the *M*-*sexta* V-ATPase in the absence (A) and presence (B) of 5mM ATP. All data were aligned to the same references and classified in Imagic-5, using the circular mask shown in the top right corner. The Scale bar represents 150Å. The full set of 100 classes from which these were extracted are shown in Figure S2.

The effect of ATP on the flexibility of the V-ATPase complex was investigated by collecting two additional data sets in the presence or absence of 5mM Mg.ATP. All data was collected at the same magnification and processed in the same manner, using the same alignment references and mask to ensure that any differences seen are related to the addition of ATP and not data processing artefacts. The resulting classes for the non-ATP sample showed the same classes as generated previously for the midgut *Manduca* sample (Figure 2E, F) with a number of classes showing flexion to a maximum angle of 30° (Figure 4A, Figure S2A). Interestingly, the addition of ATP resulted in the loss of classes that display extreme flexing of V_1_ relative to V_o_ with only classes displaying a maximum flexion of 10° being seen (Figure 4B, Figure S2B). Approximately 16% of ATP-treated particles showed this degree of flexion, comparable to that seen in particles without substrate.

### Modelling

**Figure 5:**
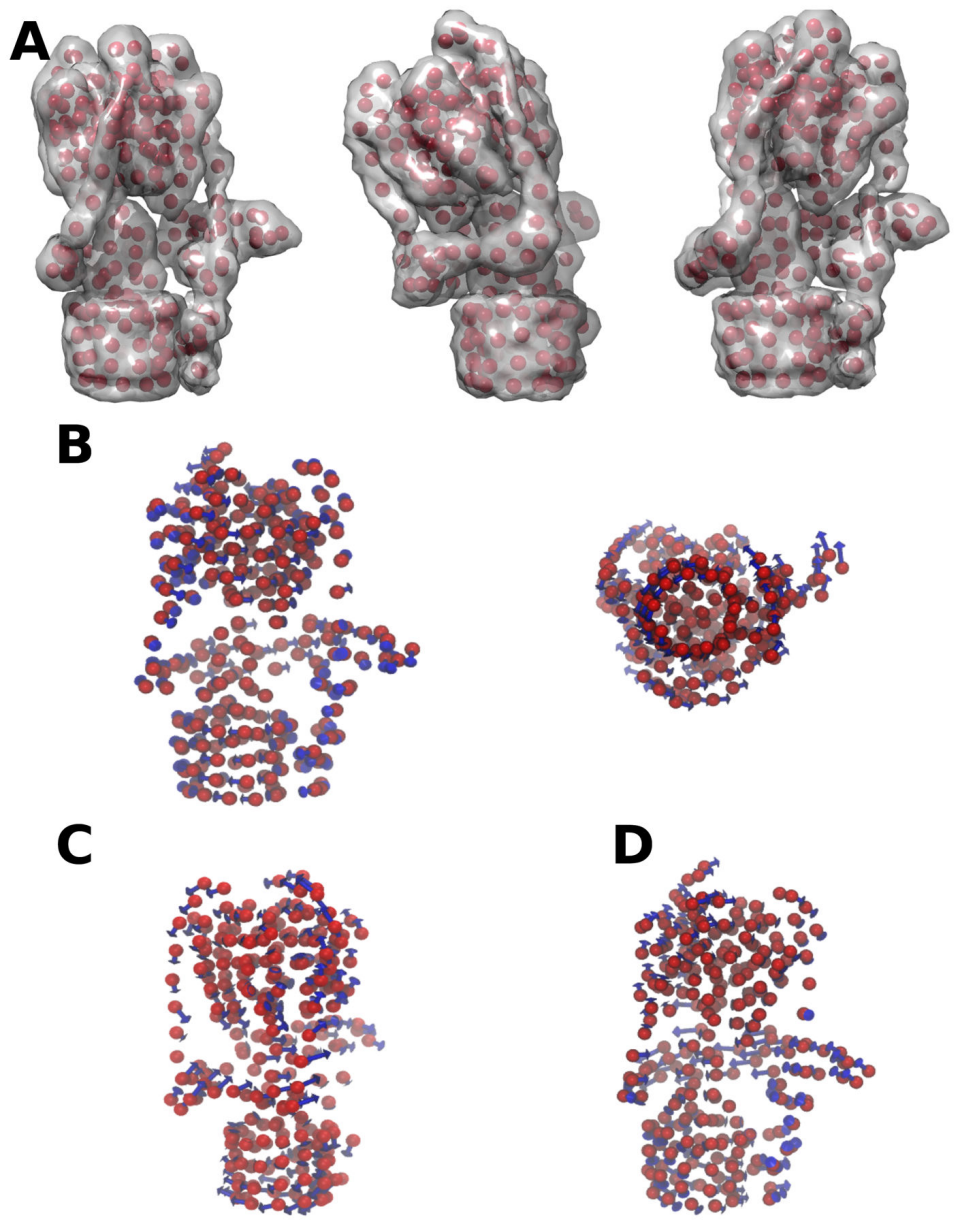
Deformation of the yeast V-ATPase along the first three non-trivial normal modes as calculated for the 256-bead ERNM. (A) Extreme conformers are depicted as a coarse-grained representation and as interpolated density maps, where the two motors are rotated against each other (left) or bended (middle, right). (B) Blue arrows represent the eigenvector corresponding to the first non-zero normal mode, which corresponds to twisting of the whole complex, consistent with the rotary mechanism of the V-ATPase. (C, D) The second and third modes of motion are bending motions with the soluble motor flexing (V_1_) with respect to the membrane rotor domain (V_o_) either back-forth (C) or side-by-side (D) suggesting that V-ATPase is laterally compliant.

The electron density of *Manduca sexta* V-ATPase was interpolated with 250 pseudo-particles (each with an average mass of ~4kDa). With such coarse-graining, application of standard elastic network models (ELN) without any ad hoc parameterization specific to the system studied is not expected to give reliable insight into the dynamics and mechanics of the complex. Instead, a bent-stretch elastic network was employed, where topological information was combined with effective, empirical force constants between the beads and employing simple arguments from linear elasticity theory. The first two non-trivial eigenmodes of the Hessian matrix of the BTS-EN model are depicted in Figure 5. The first eigenmode corresponds to the longitudinal flexing of V_1_ relative to V_o_ (Figure 5 A, B and Movies S8, S9). The second mode corresponds to a twist of V_1_ relative to V_o_ (Figure 5C and Movie S10). The eigenmodes of the model suggest that the holoenzyme is dynamic and highly deformable in specific directions along low frequency modes, which are often functionally important. These motions are largely encoded in the topology of the complex indicating they are likely to be a universal characteristic of the ATPases. 

## Discussion

Rotary ATPases have extraordinary levels of efficiency of energy transduction, far in excess of that seen in macroscopic man-made mechanical systems [56]. Intrinsic flexibility of components within the rotary ATPases is likely to be a significant contributory factor to such efficiency. Flexibility has been postulated for the F- and (by analogy) V-ATPase mechanism in two possible ways. Firstly, a spring-like function is proposed to mediate elastic power transmission between the asymmetric ATPase and H^+^ translocation motors in F-ATPase. Single molecule experiments indicate low compliance for the single peripheral stator filament in F-ATPase (i.e. low flexibility), but a highly compliant γ-subunit axle that most likely fulfils the power transmission role [57,58]. Corresponding observations have not been made with the V-ATPase, but the existence of a similar elastic function is implied by the need to satisfy the motor asymmetry also present between V_1_ and V_o_. 

The second area where flexibility in rotary ATPase structures is implicated is in accommodating the large-scale conformational changes that occur sequentially in the soluble domain during the catalytic cycle. In particular, the C-terminal region of the β subunit in F-ATPase moves closer to the membrane domain when its catalytic site adopts the ‘open’ (empty) state. This region contains the DELSEED loop that articulates against the axle as part of the process of delivering torque. The presumption is that the corresponding region in V-ATPase subunit A (containing SALSDSD or ASLAETD in *Saccharomyces* and *Manduca*, respectively) will undergo similar significant movement during the catalytic cycle. Single molecule bead reporter experiments on the F_1_-ATPase show an increase in the radius of rotation indicative of a ~4° outward tilt of the γ-subunit axle imposed by changes in conformation of the αβ unit upon binding ATP [59]. In the context of the whole F-ATPase, in which the c-ring would be constrained by the membrane and by contact with subunit-*a*, this change in rotor angle would translate into ‘wobble’ of the ATPase motor as the rotor processes through its full cycle. This wobble has to be accommodated by radial movement of the stator filament. Flexibility within F-ATPase is also suggested by crystallographic studies of F_1_-*c*_10_ complexes [26,27] where the central axle pivots at its point of contact with the *c*-ring, such that axle and *c*-ring are no longer co-axial as required for smooth power transmission. Although the ~11° flexion observed in these studies is imposed by crystal lattice interactions and likely also to be influenced by the absence of the membrane-anchored part of the stator, subunit-*a*, it is reasonable to suppose that it does report on natural flexibility. 

It is also noteworthy that in the crystal structure of the membrane extrinsic part of F-ATPase [60], the partially resolved stator filament comprising subunits OSCP/b/d/F_6_ bends towards the central axle by a greater angle than that displayed in the cryo-EM reconstruction of the whole complex [61]. This implies that in the F-ATPase the stator is ‘spring-loaded’, clamping subunit *a* onto the c-ring whilst maintaining sufficient flexibility to accommodate changes in the surface of the (αβ)_3_ complex during rotation and any eccentricity in the rotation of the c-ring. Although there are differences between A/V- and F-ATPases in their catalytic sub-steps [62], the two enzymes share enough fundamental similarities to suggest that the same ‘wobble’ should be observed for the V-ATPase [31]. On the basis of clear parallels in its organisation (reviewed in 1), flexion of inter-domain structures in V-ATPase may also be anticipated, but no analysis of this in the fully-assembled complex has been presented to date. It seems likely that this wobble and the corresponding changes to the positions of the stator filaments that accommodate it are what we observe in the E.M. data for both *Manduca* and *Saccharomyces* V-ATPases.

The stator filaments of the F and V/A-ATPases are different both in composition and number (Figure 1). The single F-ATPase has a multiple helical fold [63], whereas A- and V-ATPases contain respectively 2 and 3 right-handed coiled-coil helical filaments [11,12,15,16,63–65] only one of which may be directly linked to subunit *a* [66]. The main body of the central rotor axles of both A/V- and F-ATPases are similar in that they contain an extended helical coiled-coil [67,68], but differ significantly in both size and composition in the region that interacts with the *c*-ring. In A- or V-ATPases, the subunit DF axle may interact with the *c*-ring only indirectly via the *C* or *d* subunits, respectively (see Figure 1) and may therefore have different mechanical properties compared to its F-ATPase equivalent. Although the limited resolution of the EM classes makes it difficult to decide, our data are more consistent with a change in the angle at which the axle exits the (AB)_3_ headgroup rather than articulation at the D/*d*/*c*-ring coupling.

 Unlike the F-ATPase, there are no crystal structures for the V_1_/A_1_c_10_ complex but there are a number of electron microscopy 3D reconstructions. A likely reason for flexibility not being directly observed in these is that any particles showing any significant deviation from the global average will be removed during image processing. ‘Misaligned’ particles will include those displaying the greatest degree of flexing. By focusing on the particles which have previously been removed from a data set, we have shown that the V-ATPase is able to flex about its central axis by up to 30°. This is greater than the (maximally) ~11° seen in F_1_-c_10_ crystal structures, and the radial bending proposed for the A-ATPase stator filament based on variations in crystal structures of the subunit EG heterodimer and normal mode analysis (7°) [31]. 

**Figure 6:**
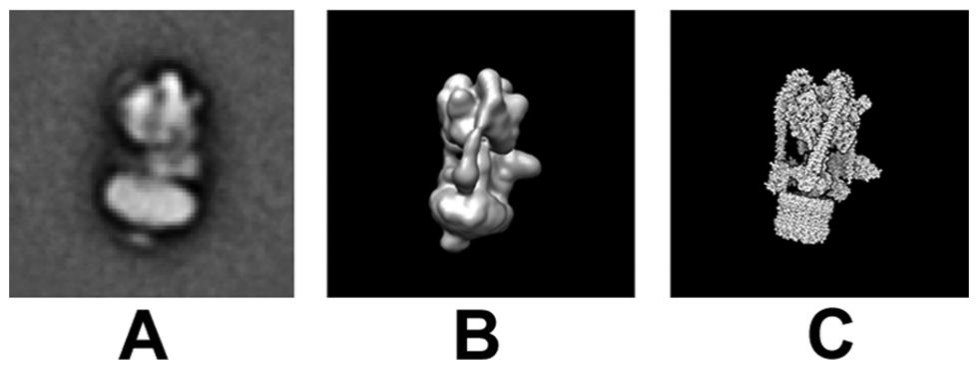
Comparisons between the NMA models and negative stain classes. (**A**) Negative stain class of *M*. *sexta* V-ATPase showing the maximum flexing between V_1_ and V_o_ relative to each other. A view of the molecular dynamic simulation in the most “flexed” state of *M*. *sexta* V-ATPase as a map representation (B) and atom representation (C) shown in the same orientation as (A) with the equivalent flexing of V_1_ relative to V_o_.

 The absence of complete molecular models for any rotary ATPase, in particular for the membrane bound subunit-*a*, prevents the application of standard atomistic modelling and molecular simulation methodology. However, topology and shape are often sufficient to predict the dynamic behaviour of proteins and their complexes [69]. A successful and popular approach to coarse-graining is the application of Elastic Network Models (ENM), used in a variety of biophysical and structural biology-related problems. In the absence of atomistic resolution models for the V-ATPase, ENMs (or variants, as in this case) can be applied not to real atoms, but to pseudo-atoms representing the distribution of electron density observed by cryo-EM. The resulting simulations of the V-ATPase show two low frequency modes (Figure 5) – higher-frequency modes of coarse-grained models are not expected to be trustworthy, so have been discarded here. The first of the reliable models (Movies S8 and S9) shows flexion along the long axis of the complex, consistent with the stator ‘wobble’ evident from the EM data analysis (Figure 6). 

Why is flexing visible within an ATP-depleted V-ATPase population? V-ATPase reconstructions of yeast and *Manduca* V-ATPase have both been shown to adopt a defined ‘resting’ state characterised by the projection of one A subunit closer to the membrane domain [63]. This A subunit (between stator filaments S1 and S3, as defined in [1]) is most likely part of the catalytic unit in the ‘open’ nucleotide-free state. The equivalent F_1_-ATPase β subunit has been shown by high speed atomic force microscopy to project in a similar way [24]. What drives the complex into this uniform resting state is unclear, but differences in conformation of one or more stator could be a factor, as could asymmetry in the *c*-ring [63]. The observation of flexion in the EM datasets could be explained if not all of the V-ATPase particles were able to return to the resting state. Since V_1_ has three catalytic sites, the V-ATPase must in some cases turn over a further 2 ATP molecules before reaching the resting state, which will not always be possible as ATP is depleted. The resting state is likely the predominant species within a V-ATPase sample population and will therefore dominate image processing. Normally, particles not in the common resting state will show a subtle change in structure which will result in them being removed in the final reconstructions. The ability to clearly define a resting state within the current V-ATPase reconstructions, shows this to be the case as a mixed population of states in the V_1_ domain would result in an apparent 3 fold symmetric structure representing the average V_1_ domain and not independent states. By examining the entire dataset, we are able to see the small proportion of particles trapped in an as-yet undefined state in which the axle is ‘cocked’, comparable to the F_1_-ATPase ‘catalytic dwell’ [59]. We cannot rule out that the longitudinal flexing that we see in both the EM analysis and normal-mode analysis could have a Brownian component.

It is also important to consider the role that the longitudal flexing motion may play in the regulatory mechanism of the V-ATPase. Both the yeast and *Manduca* V-ATPase have been shown to be regulated through controlled dissociation, whereby the V_1_ domain separates from the V_o_ domain through a series of currently unresolved structural changes, but likely effected through changes at the subunit *a*/C/E/G interface [37]. Importantly, the addition of Mg.ATP substrate does not increase either the range of the flexion or the proportion of particles that are flexed. Instead, it limits the flexibility to a maximum of 10° (Figure 2B, Figure 4B, Movies S1-S3, S5), the angle that is most consistent with the proposed radial bending and observed variations in crystal structures of the subunit E/G heterodimer [26,31]. This more subtle movement (shown in Figure 2B, C and Movies S1-S3) is also consistent with the predicted flexibility within the V-ATPase [31]. The larger bending motion which is seen in Figure 2D and F and Movie S4 is more consistent with the large angle flexing seen in the early stage proposed to immediately precede disassembly of the *Thermus thermophilus* A-ATPase [38]. The apparent flexing seen for the V-ATPase could represent a snapshot of the first stage in the dissociation process since ATP, which is required for dissociation, is limiting in the medium [70]. This has important implications for crystallographic experiments on the rotary ATPase family as priming the sample with ATP may allow for a more homogeneous sample.

The normal-mode analysis of the ENM also suggests twisting of V_1_ relative to V_o_ is possible (Figure 5C, Movie S10). Since both motors work in a rotary fashion, this motion maybe more representative of an elastic storage mechanism whereby the torsional forces created in V_1_ rotate it away from V_o_, with the stators and central axle twisting in response. In principle, this motion could represent stator filament bending as part of an elastic power transmission mechanism, whereby the torsional forces created in V_1_ cause counter-rotation with respect to V_o_, with the central axle and stators, twisting in response. Although twisting motions are suggested (Movies S3 and S10), the techniques used in our EM analysis and the resolution afforded makes this movement difficult to reliably capture. While simple, shape-consistent elastic models capture the topological contribution of subunit organization, intrinsic mechanics of subunits and protein-protein interactions give rise to more complex mechanical behaviour and we expect that future development of more accurate models combined with experimental data will provide further insights. 

 Flexibility in rotary ATPases has been predicted through incomplete crystal structures and molecular dynamic simulations of components of the complex. Here we show flexibility in V-ATPase, using molecular dynamic simulations and electron microscopy, with both flexing motion of V_1_ relative to V_o_ to a maximum of 30° and rotation of the two domains relative to each other (Figures 2, 5 and 6). Such flexibility has implications for elastic transmission and the dissociation mechanism. Future work will be required to distinguish if longitudinal flexing results from Brownian forces on the complex, with the twisting motion seen in the system contributing to the rotational mechanism. Mechanical distortions in rotary ATPases are likely to be crucial elements of their mechanisms but are only just starting to be explored.

## Supporting Information

Figure S1 **Classification tests to check for artifacts in data processing.** (A) The *M. sexta* V-ATPase negative stain data set aligned and classified using the full data set and a full mask (top left corner), representatively flexed classes were extracted and shown. (B) The *M. sexta* V-ATPase negative stain data set with those particles which aligned to a specific view extracted and then re-aligned and classified using a full mask. (C) The *M. sexta* V-ATPase negative stain data set with those particles which aligned to a specific view extracted and then re-aligned and classified using a mask which only covered V_o_. Despite the three different processing routes for A-C similar classes are obtained. (D) The Yeast V-ATPase negative stain data set classified using a full mask and those classes which displayed large flexing extracted. The Scale bar represents 150Å.
(TIF)

Figure S2 **Classes of *M. sexta* V-ATPase in the presence and absence of ATP.** Negative stain analysis of the *M. sexta* Malpighian tubule V-ATPase in the absence (A) and presence of 5mM ATP (B). All data were processed using the same procedures, alignment references and mask. The aligned stacks were then classified into 100 representative classes using Imagic-5 [46]. Those classes which display significant flexing of V_1_ relative to V_o_ are shown by a star. The scale bar represents 20nm.
(TIF)

Movie S1 **Yeast V-ATPase flexibility about V_1_.** This movie shows ~30° flexing of the V_1_ domain relative to a fixed V_o_ domain. The data is generated from negative stain classes.
(AVI)

Movie S2 **Yeast V-ATPase flexibility about V_o_.** This movie shows flexing of the V_o_ domain relative to a fixed V_1_ domain. The data is generated from negative stain classes.
(AVI)

Movie S3 **Yeast V-ATPase central flexibility.** A movie to show the apparent flexibility about the central region of the yeast V-ATPase. The data is generated from negative stain classes.
(AVI)

Movie S4 ***M. sexta* V-ATPase flexibility about V_0_.** This movie shows ~30° flexing of the V_o_ domain relative to a fixed V_1_ domain of *M. sexta* V-ATPase. The data is generated from negative stain classes.
(MPG)

Movie S5 **Further *M. sexta* V-ATPase flexibility about V_o_.** This movie shows flexing of the V_o_ domain relative to a fixed V_1_ domain at a view ~90° to that of movie S4 with subunit *a* facing the viewer in the right hand side. The data is generated from negative stain classes.
(MPG)

Movie S6 ***M. sexta* flexibility within the Cryo-EM data set.** A movie to show the apparent central stalk rotation within the *M. sexta* V-ATPase resulting in the flexing of the V_o_ domain.
(MPG)

Movie S7 ***M. sexta* flexibility of V_o_ relative to V_1_.** Flexing of the V_o_ domain relative to the V_1_ domain as seen in the Cryo-EM *M. sexta* data set.
(MPG)

Movie S8 **Molecular dynamic simulation of the flexibility within *M. sexta* V-ATPase.** A movie to show the flexibility calculated for *M. sexta* V-ATPase based on a molecular dynamic BTS-EN approach. *M. sexta* V-ATPase is seen from an equivalent view as Movie S4.
(MPG)

Movie S9 **Alternative view of the calculated flexibility within *M. sexta* V-ATPase.** This movie shows the calculated flexibility for *M. sexta* V-ATPase based on a molecular dynamic BTS-EN approach. *M. sexta* V-ATPase is seen from an equivalent view as Movie S5.
(MPG)

Movie S10 **Molecular dynamic simulation of the domain twisting within *M. sexta* V-ATPase.** A movie to show the apparent twisting of the V_1_ domain relative to the V_o_ domain calculated by a molecular dynamic BTS-EN approach. *M. sexta* V-ATPase is seen from an equivalent view as Movies S5 and S9.
(AVI)
