# Supplementary figures and images for: Flexibility within the Rotor and Stators of the Vacuolar H+-ATPase
Source: PLoS One. 2013 Dec 2;8(12):e82207. doi: 10.1371/journal.pone.0082207 (PMC3846802; doi:10.1371/journal.pone.0082207)

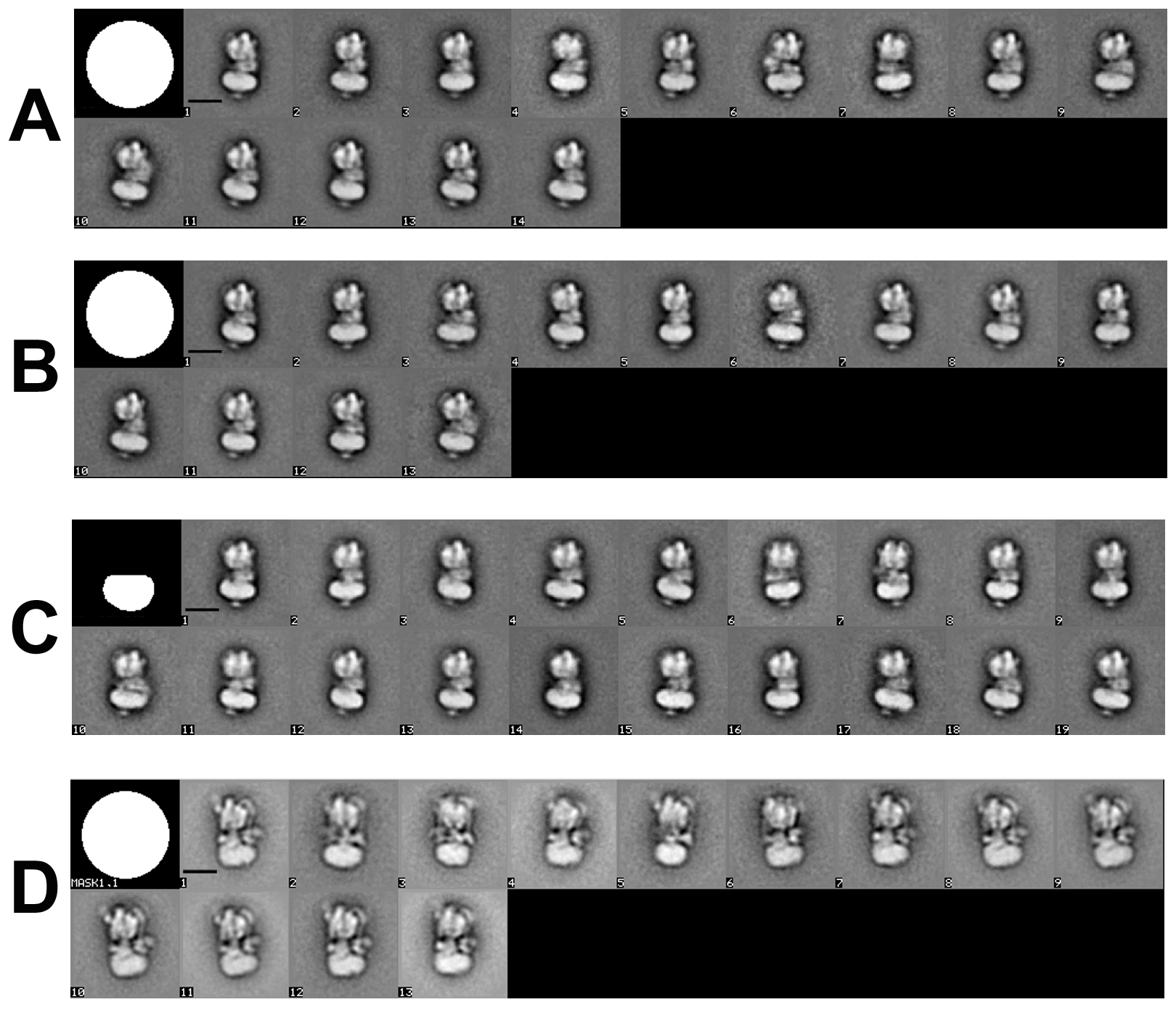

Supplement: Figure S1 — Classification tests to check for artifacts in data processing. (A) The M. sexta V-ATPase negative stain data set aligned and classified using the full data set and a full mask (top left corner), representatively flexed classes were extracted and shown. (B) The M. sexta V-ATPase negative stain data set with those particles which aligned to a specific view extracted and then re-aligned and classified using a full mask. (C) The M. sexta V-ATPase negative stain data set with those particles which aligned to a specific view extracted and then re-aligned and classified using a mask which only covered Vo. Despite the three different processing routes for A-C similar classes are obtained. (D) The Yeast V-ATPase negative stain data set classified using a full mask and those classes which displayed large flexing extracted. The Scale bar represents 150Å. (TIF) [file pone.0082207.s001.tif]

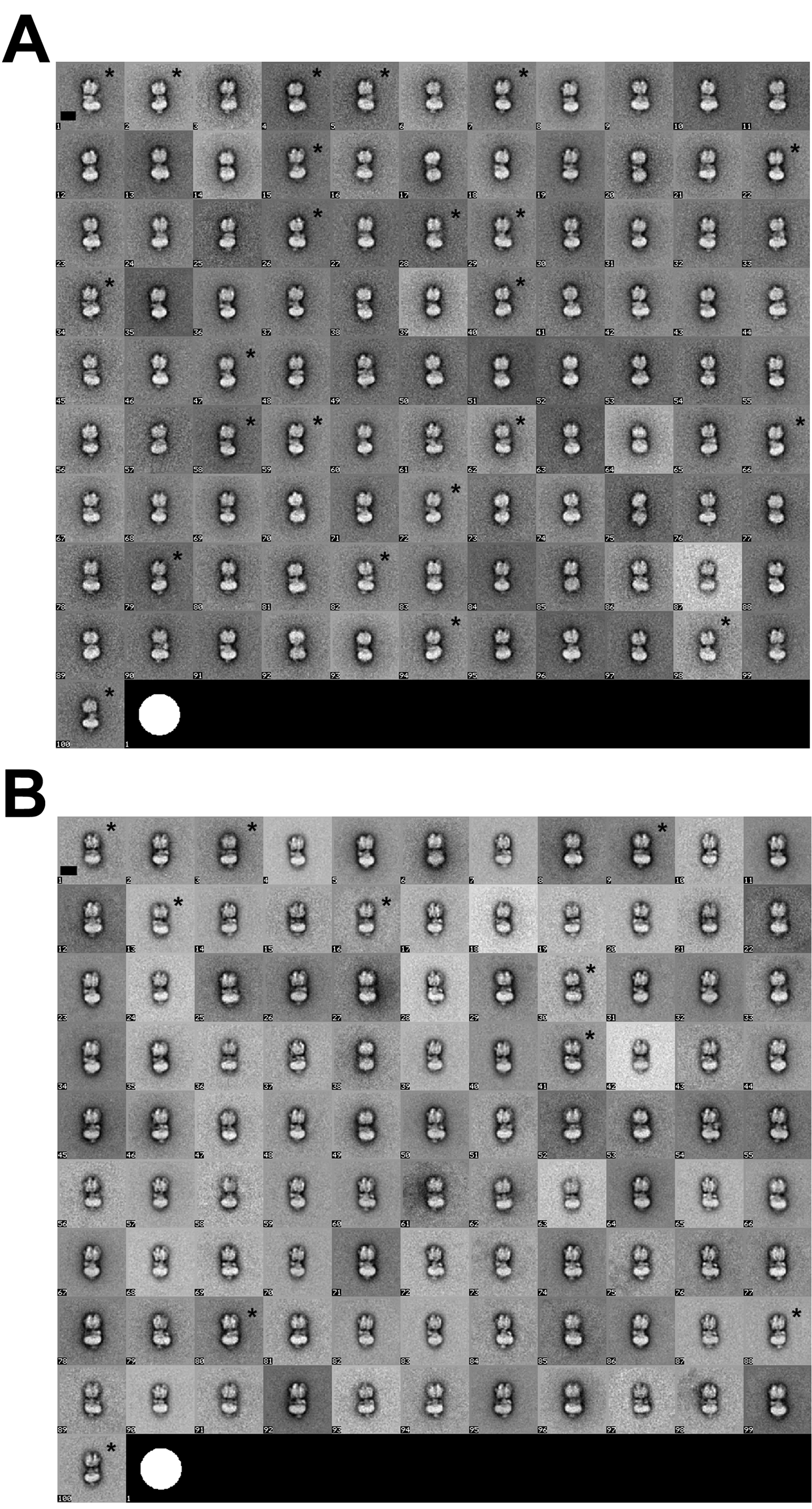

Supplement: Figure S2 — Classes of M. sexta V-ATPase in the presence and absence of ATP. Negative stain analysis of the M. sexta Malpighian tubule V-ATPase in the absence (A) and presence of 5mM ATP (B). All data were processed using the same procedures, alignment references and mask. The aligned stacks were then classified into 100 representative classes using Imagic-5 [46]. Those classes which display significant flexing of V1 relative to Vo are shown by a star. The scale bar represents 20nm. (TIF) [file pone.0082207.s002.tif]
